# Lower Affinity T Cells are Critical Components and Active Participants of the Immune Response

**DOI:** 10.3389/fimmu.2015.00468

**Published:** 2015-09-10

**Authors:** Ryan J. Martinez, Brian D. Evavold

**Affiliations:** ^1^Department of Microbiology and Immunology, Emory University, Atlanta, GA, USA

**Keywords:** TCR affinity, 2D assays, tetramers, T cells, T cell diversity

## Abstract

Kinetic and biophysical parameters of T cell receptor (TCR) and peptide:MHC (pMHC) interaction define intrinsic factors required for T cell activation and differentiation. Although receptor ligand kinetics are somewhat cumbersome to assess experimentally, TCR:pMHC affinity has been shown to predict peripheral T cell functionality and potential for forming memory. Multimeric forms of pMHC monomers have often been used to provide an indirect readout of higher affinity T cells due to their availability and ease of use while allowing simultaneous definition of other functional and phenotypic characteristics. However, multimeric pMHC reagents have introduced a bias that underestimates the lower affinity components contained in the highly diverse TCR repertoires of all polyclonal T cell responses. Advances in the identification of lower affinity cells have led to the examination of these cells and their contribution to the immune response. In this review, we discuss the identification of high- vs. low-affinity T cells as well as their attributed signaling and functional differences. Lastly, mechanisms are discussed that maintain a diverse range of low- and high-affinity T cells.

## Introduction

The T cell immune response is composed of diverse sets of T cell receptors with a normally distributed range of affinities for pMHC (1, 2). Upon antigen recognition, the biophysical characteristics of the TCR:pMHC interaction will drive signaling, division, and differentiation (3, 4). Current hypotheses suggest the highest affinity T cells have a competitive advantage during the immune response, based on the assumption that they would receive stronger and more prolonged activation signals than T cells with lower affinity interactions (5–7). However, evidence is emerging to suggest the lack of skewing toward the highest affinity T cell repertoire, instead of proposing a distribution of affinities and maintenance of diversity throughout the immune response (8, 9). This T cell diversity has been shown to be important for homeostasis of the immune system, but the process of maintaining affinity diversity is unclear. We further discuss factors that support and favor the survival of lower affinity T cells and, therefore, highlight the important contribution of low-affinity T cells and a broad affinity distribution in the immune response.

## Measurement of TCR:pMHC Affinity

Affinity is defined as the probability of a receptor (TCR)–ligand (pMHC) interaction and is one of the most commonly used measurements to predict the T cell response to antigen. As with all receptor–ligand interactions, affinity, or the association constant (*K*_a_) is derived from the on-rate (*k*_on_) and off-rate (*k*_off_) of receptor–ligand equilibrium and is calculated independently of concentrations of ligand and receptor (affinity = *K*_a_ = *k*_on_/*k*_off_) (Table 1) (3, 10). The affinity of an interaction is not its bond strength, or the force needed to break the bond, though these terms are often incorrectly used interchangeably. An example of this distinction can be seen in the avidin/biotin system as this receptor–ligand pair has one of the highest affinity interactions measured, but the non-covalent interactions can be easily broken, signifying lower bond strength (11). Along with TCR:pMHC affinity, the dissociation constant (*K*_d_, inverse of *K*_a_) and half-life [τ_1/2_ = ln(2)/*k*_off_] can also be derived. The combination of these measurements have been used to describe T cell activation and differentiation models (kinetic proofreading, optimal dwell time, etc.), but not all methods of biophysical measurements generate similar results (3, 12, 13). Therefore, further understanding of measurement systems is necessary.

**Table 1:** **Definitions of biophysical parameters of TCR:pMHC interaction**.

Keyword	Definition
Affinity	Tendency of association between a monovalent receptor–ligand pair at equilibrium. Two types of affinity measurements can be appreciated
	*Absolute affinity*: affinity measurement using purified receptor and ligand without cellular context
	*Relative affinity*: affinity measurement of receptor–ligand interactions in cellular membranes to recapitulate native interactions
Avidity	Tendency of association between multivalent entities at equilibrium. This is not the sum of affinities as the binding of one receptor–ligand pair increases the likelihood of another receptor–ligand pair interaction due to reduced proximity and degrees of freedom
Association constant (*K*_a_)	The equilibrium constant of the association of a receptor and ligand
Dissociation constant (*K*_d_)	The equilibrium constant of the dissociation of a receptor–ligand interaction
On-rate (*k*_on_)	The rate at which a receptor and ligand bind to form a complex
Off-rate (*k*_off_)	The rate at which a receptor–ligand complex reverses binding
Half-life (τ_1/2_)	The amount of time needed for half of the receptor–ligand complexes to reverse binding

Affinity is commonly measured using purified reactants in solution by surface plasmon resonance (SPR), where free ligand is flowed over receptors fixed to a surface. This absolute affinity measurement occurs in three dimensions (3D) and allows for the definition of protein interactions in their simplest, purified form with no influence from outside forces. However, the increased degrees of freedom that occur in solution may not be the optimal method to accurately assess interactions between receptor and ligands that occur in two opposing plasma membranes. A better replicate of *in vivo* interactions between proteins at the membrane surface can be accomplished using two-dimensional (2D) receptor–ligand binding techniques, such as flow chamber assays, thermal fluctuation assays, single molecule FRET, Zhu–Golan plots, contact area FRAP, and the adhesion frequency assay (3). Currently, the focus of our lab has been the use of the two-dimensional micropipette adhesion frequency assay (2D-MP), a measurement of the relative 2D affinity of the receptor–ligand interaction on opposing membranes (14). This 2D affinity is termed a relative affinity because it is dependent on the context in which it was measured, whereas 3D methods generate an absolute affinity measurement while ignoring all other cellular participants. This distinction of relative and absolute affinity will be discussed in a later section. When 2D and 3D affinity TCR measurements are compared, an increased affinity with an associated decreased *k*_off_ can be appreciated (12, 13, 15, 16). Attempts to correlate affinity values generated by 2D and 3D methods have been achieved with little success as the parameters controlling relative 2D affinity are still unknown (12). Importantly, the relative affinity measured by 2D-MP better correlates with functional responses than 3D methods and refers to the affinity in the proper cellular context (12, 15).

The advent of recombinant pMHC tetramer reagents has allowed for the identification of antigen-specific T cells and the subsequent use of these reagents for indirect assessment of biophysical interactions of TCR:pMHC. The binding of the tetramer reagent is dependent on valency to increase its avidity as monomeric pMHC complexes do not attach well to TCR (17, 18). This lack of monomer interaction with TCR is most likely due to the reliance of pMHC tetramer staining on higher affinity interactions (8, 9). The *k*_off_ and *k*_on_ for each arm of the pMHC tetramer binding to TCRs are known to reflect avidity interactions, with the binding of one pMHC monomer arm enhancing the *k*_on_ of the subsequent monomer arm and reducing the *k*_off_ of the entire reagent (19). The use of pMHC tetramer to measure *k*_off_, *k*_on_, and τ_1/2_ assumes that the amount of pMHC tetramer bound to a cell is directly proportional to the affinity of that cell, with more tetramer bound to higher affinity cells than to lower affinity T cells (6, 9, 19, 20). However, this assumption may not always yield a direct correlation, with many groups demonstrating tetramer binding intensity does not equate to functional responses or SPR measurements (21–24). One possible explanation for discrepancies with SPR is that the cellular membrane can affect tetramer binding. Another possibility for these discrepancies is that TCR density affects binding because tetramer relies on avidity interactions. While many have normalized the TCR to pMHC concentrations on each cell (18, 25, 26), others do not account for the number of TCRs expressed at the cell surface (21, 27, 28). The effect of TCR density can be appreciated as the analysis of the tetramer+ populations reveals lower TCR expression as they exhibit only 20–40% of the TCR density compared to the bulk T cell population (unpublished data). This indicates tetramer+ T cells may have different TCR levels than the remaining T cell population but it is unknown if this is a cause or an effect of being a tetramer binder.

The measurement of TCR:pMHC affinity by 2D-MP is an extremely sensitive method that follows first-order kinetics and is dependent upon T cell intrinsic factors (3). Measured TCR affinities can be altered when reagents are used to change lipid composition and actin cytoskeleton (12). Adjustments of the membrane and supporting scaffolding should change 2D affinity as the characteristics of the opposing membranes during receptor–ligand interactions are fundamental for the measurement of relative 2D affinities. Much of the sensitivity of the 2D-MP assay comes from the flexibility of the red blood cell (RBC) membrane, which can be distended by the formation of a single TCR:pMHC bond (3, 29). As biotinylated pMHC is bound to the RBC through streptavidin interactions, clustering and valency of ligand could play a role in binding. Varying the concentration of pMHC on the RBC surface does not change the calculated affinity of activated T cells, signifying concentration does not affect the 2D measurements (12, 14). In experiments altering the valency of pMHC on the RBC through use of mutant streptavidin, no changes in 2D affinity were noted (12). This is in contrast to pMHC tetramers, which rely on both concentration and valency of the reagent to measure antigen specificity (30). Together, this suggests concentration and valency do not play a role in the measurement of affinity by 2D-MP. In addition, it demonstrates the micropipette values are a measure of affinity and not avidity.

An important distinction between CD4 and CD8 T cells is the contribution of coreceptor to the overall strength of binding between pMHC and TCR (18, 31). CD4 and CD8 coreceptors are thought to stabilize TCR:pMHC bonds while also recruiting Lck to the TCR complex for the initiation of the downstream signaling cascade (32, 33). Intriguingly, CD8 has a higher affinity for its coreceptor than CD4, though both interactions are of weaker affinity (13, 32). For CD8 T cells, coreceptor contributes to the binding of TCR to pMHC when assessed by pMHC tetramer, 2D-MP, and SPR (31, 34–36). The removal of CD8 contribution leads to decreased avidity and functional responses (37–39). In addition, the binding of the lowest affinity T cells are the most affected by the loss of CD8, signifying CD8 helps to increase the likelihood of low-affinity TCR:pMHC interaction and signaling (15, 37). In contrast, the role of CD4 is very different as there is little to no contribution to the TCR:pMHC interaction as measured by 2D-MP, pMHC tetramer, and SPR (13, 32). This is not to say CD4 is not important in functional responses as CD4 is required to recruit Lck for optimal initiation of T cell signaling (17, 40, 41), but at least under the conditions used in tetramer and 2D assays, there is little contribution to biophysical parameters (13). Importantly, detection of CD4 T cells by pMHCII tetramer would be expected to miss more of the lower affinity TCRs due to the lack of coreceptor contribution as compared to pMHCI tetramer and CD8 T cells. These differences in CD4 and CD8 coreceptor binding impact the use of tetramers to count antigen-specific T cells as well as measuring the kinetic rates of the TCR:pMHC interaction.

## Detection of Antigen-Specific T Cells

The identification of T cells is important as the biophysical TCR:pMHC interactions discussed are correlated with functional responses. The current gold standard for identifying antigen-specific T cells are using either pMHC tetramer reagents or readouts of functional responses, but both are sub-optimal in identifying the true number of antigen-specific T cells (42–45). For example, not all T cells of the same specificity make cytokine upon stimulation as demonstrated by the use of TCR-Tg T cells (46, 47). Further, a CD4 T population has a number of distinct fates with associated effector functions, such that any one cytokine will underestimate the total number of antigen-specific cells. Interestingly, the number of pMHC tetramer+ T cells sometimes equates to the number of cytokine-producing cells, even though not all T cells will produce the target cytokine (27, 43, 45). For the most part, intracellular staining for cytokines is incompatible with tetramer staining making it unclear if cytokine-producing cells overlap with tetramer binders, or if they are distinct populations. To more accurately identify the number of antigen-specific T cells, groups have used activation markers, such as CD11a, LFA-1, or CD49d, that are upregulated on antigen-specific T cells after infection (43, 48). When these cells are quantitated using these functional cell-surface markers, they far outnumber the number of tetramer or cytokine-producing cells (43, 48). The 2D-MP further corroborates the underestimation of antigen-specific T cells by pMHC tetramers (8, 9). When compared to the total population of antigen-specific T cells, pMHC tetramer+ T cells make up only the highest affinity population (9). Most T cell repertoires have a normally distributed TCR:pMHC affinity range, with the rarest cells being the highest and lowest affinity. Based on this data, tetramer+ cells are above average in affinity and would, therefore, only make up a fraction of the total T cells in an immune response (4). This would be especially apparent for CD4 T cells since the coreceptor does not aid in tetramer avidity.

**Figure 1:**
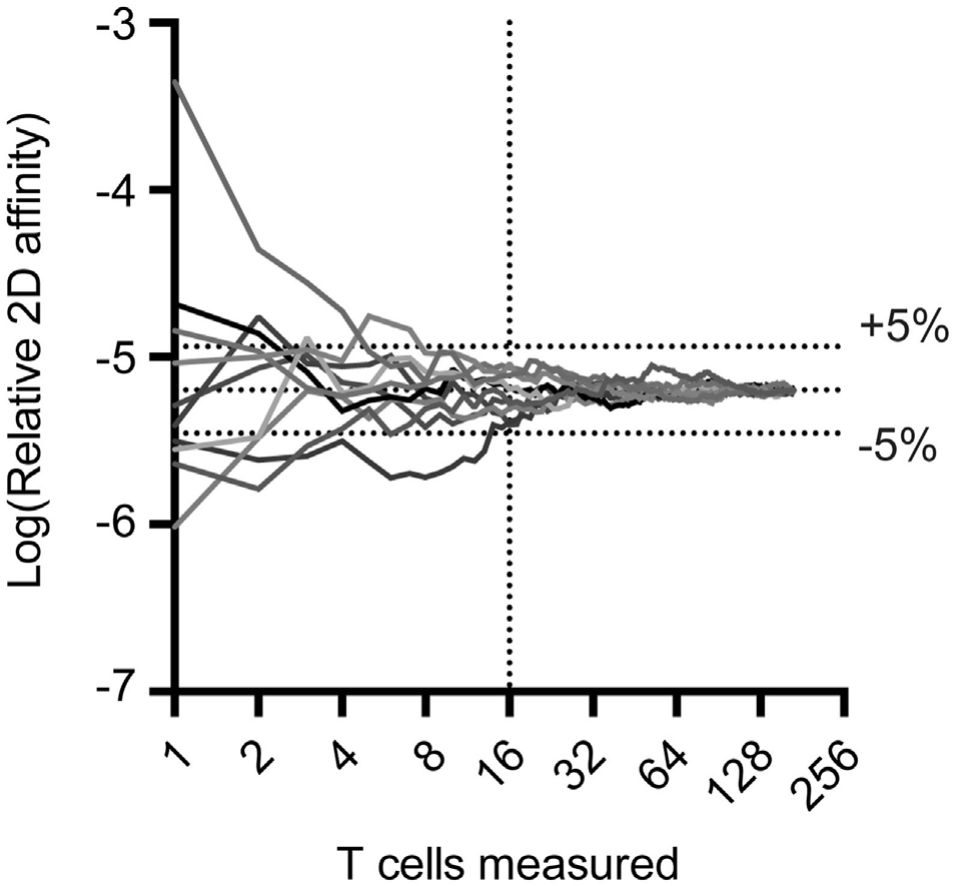
**Random sampling of measured TCR:pMHC reveals rapid approach to average affinity for an entire population**. Previous 2D affinity measurements of MOG-specific CD4 T cells were combined and randomly arranged. A moving average was calculated and graphed as a function of the number of cells sampled. Cells were then randomly rearranged and moving average calculations were performed nine more times to generate the curves illustrated.

The 2D-MP assay is currently the most sensitive available to capture the entire affinity range of a T cell repertoire and not just the highest affinity T cells. To perform the 2D-MP assay, single T cells are randomly chosen for affinity measurements from a purified population of cells. As this is a sampling process that is time intensive, it is important to understand the numbers of cells needed for analysis to reflect representative data of the entire population. To address how many cells need to be measured to find the average affinity of an antigen-specific polyclonal population, we have combined previously published measurements of a polyclonal T cell population for a single antigen (MOG_38–49_:I-A^b^) and performed random sampling experiments (Figure 1). When noting the moving average from 10 repetitions of the randomly sampled MOG-specific T cell affinities, it is apparent that the sampling affinity often reaches the average affinity rapidly, i.e., within 10 binding pairs. By the time, 16 binding T cells have been analyzed, the average affinity measured is ±5% of the affinity of the entire pooled population. This measurement is for a polyclonal population with a 2,000-fold range in affinity, meaning a repertoire with considerably less diversity (TCR-Tg or tetramer+ T cells) needs even fewer points measured to identify the average affinity. Therefore, to measure the average affinity of a normally distributed polyclonal repertoire only 10–16 T cell affinities need to be measured to be within 5% of the average affinity of the repertoire.

## Signaling in Low-Affinity T Cells

In both kinetic-segregation and proofreading models, the binding of TCR:pMHC is key to activation, with the *k*_off_ and *k*_on_ and τ_1/2_ controlling the strength of signal the T cell receives (49–53). Higher affinity interactions with prolonged τ_1/2_ have an increased likelihood of forming a stable conjugate and triggering the T cell signaling cascade, though an optimal τ_1/2_ most likely exists due to the hypothesized need for serial engagement (10, 50, 53). Using this logic, the lowest affinity T cells should have a reduced probability of initiating T cell signaling, but these cells do efficiently propagate the signaling cascade as low-affinity T cells do expand, and differentiate during the immune response (8, 9, 48). Thus, mechanisms must exist that allow for low-affinity T cells, with the reduced probability of initial TCR:pMHC bond formation, to receive sufficient signals to compete with high-affinity counterparts.

Developing thymocytes discriminate positive- and negative-selecting signals through both qualitative and quantitative signaling pathways. Qualitatively, studies have suggested that reduced but sustained Erk signaling is necessary for positive selection, while strong Erk, p38, and Jnk activation are necessary for negative selection (54–57). In positive selection, the protein Themis reduces TCR signaling by recruiting the phosphatase SHP-1 to inhibit Lck activation and reduces strong, transient, Erk activation, a property associated with negative selection (57, 58). Without Themis, low-affinity pMHC can produce strong agonist signals resulting in increased negative selection (58). This is of interest as negative regulatory mechanisms must be preventing low-affinity ligands from being selected like high-affinity ligands. Currently, it is unknown what controls the function of Themis or how it discriminates between high- and low-affinity pMHC to ultimately regulate downstream signals. One potential quantitative mechanism for discriminating high- and low-affinity pMHC was found in thymocyte negative selection. During negative selection, thymocytes bind to pMHC and scan coreceptors (CD4 and CD8) to find one coupled to Lck (59). CD8 coreceptors on thymocytes have a lower amount of coupled Lck than CD4 coreceptor, so a longer TCR:pMHC τ_1/2_ is necessary for CD8 T cell negative selection (59). If a TCR:pMHC bond is maintained during the duration for a coreceptor-Lck conjugate to be found, the T cell is determined to be high affinity and will undergo Bim-dependent apoptosis (60). These differences in signaling between positive and negative selection demonstrate how similar stimuli (pMHC) can generate distinct results depending on the TCR interaction parameters.

Naive CD4 and CD8 T cells demonstrate a distribution of reactivity to multiple foreign antigens that is established during thymic selection on self-antigens. In both naïve CD4 and CD8 T cells, multiple studies have been able to identify heterogeneity in reactivity of T cells for self- and foreign-pMHC (26, 61–64). These ranges of reactivity relate to markers of self-pMHC stimulation (CD5, Nur77, basal TCRζ phosphorylation) (26, 61, 64, 65) as well as indicators of activation in response to foreign-pMHC (ERK phosphorylation, IL-2 production) (64). Conflicting experimental evidence exists when attempting to correlate self-reactivity with foreign-reactivity (26, 61, 64). Some have demonstrated that CD5 positively correlates with foreign TCR reactivity (measured by pMHC tetramer and function) (26, 61), while others have demonstrated CD5 expression can be altered without changes to TCR affinity as measured by SPR (64). Nonetheless, based on the TCR affinity for self-pMHC, the immune system is able to diversify the reactivity of signaling machinery by controlling the basal phosphatase and kinase activity.

The first step of signaling after TCR triggering is phosphorylation of CD3 and TCRζ intracellular chains by Lck and the recruitment of the kinase Zap70. Phosphorylation of Zap70 by Lck propagates the initial signals and causes downstream activation of the MAP kinase (Erk), NF-κB, and calcineurin pathways (66). To inhibit signaling cascades, phosphatases, such as SHP-1/2 and CD45, prevent the sustained activation of kinases (66). For low-affinity TCR:pMHC interactions, the peak of T cell signaling molecules (Erk, Jun, and Ras) are delayed or absent and TCR signaling is decreased (58, 62, 67). This late and reduced activation of Erk in low-affinity T cells allows for the sustained recruitment and activity of SHP-1, decreasing the activation of Lck and reducing the T cell signaling response (58, 67, 68). Another phosphatase, PTPN22, has also been shown to inhibit Erk phosphorylation in T cells stimulated with low-affinity pMHC (69). It is surprising that even with these negative signaling events, low-affinity TCRs are able to signal and even upregulate activation genes CD69 and CD25 to similar levels as higher affinity T cells (7, 67). Similar to thymocyte selection, this demonstrates peripheral T cells have mechanisms to detect biophysical TCR interactions that can then be translated to signaling cascades as well as possess compensatory mechanisms to enhance low-affinity TCR interactions or even decrease higher affinity T cell interactions (70). Potentially, force generation by TCRs could be a mechanism by which low-affinity T cells are able to increase bond τ_1/2_ and receive similar signals as high-affinity counterparts (71–73). Conversely, it may be that different pathways are induced by low-affinity TCR:pMHC binding that are currently not understood. Overall, low- and high-affinity T cells use similar mechanisms and machinery to signal, but the outcomes seem to vary in their response.

## Functionality of Low-Affinity T Cells

The development and function of T cells has been highly associated with TCR:pMHC affinity, with high- and low-affinity T cell interactions often performing different roles. Thymocyte selection is dependent upon TCR:pMHC affinity interactions, with positive selection requiring low-affinity interactions and negative selection deleting thymocytes greater than a threshold affinity (1, 74). The number of antigen-specific T cells for a single epitope is tightly controlled by central tolerance and, therefore, by TCRs affinity for self-pMHC presented in the thymus (75–79). The initial numbers of DP thymocytes for a single epitope are defined by the size of their positively selecting niche of self-peptides and further culled by their reactivity to self-antigen via negative selection (27, 63, 79–81). Work suggests positive selection is important in the generation of a functional T cell repertoire as alterations in the size of the selecting niche can cause T cell dysfunction or increased reactivity to antigen (63, 82). Analysis of the autoimmune prone NOD mouse has suggested its positive selecting niche is reduced, causing competition for positive selection survival signals and enriching for T cells that have a higher affinity TCR:self-pMHC interaction (81). This reduced positive selection niche and concurrent increase in TCR affinity will not be fully compensated by negative selection as negative selection is not as effective as once believed (63, 81, 83–85). The precursor frequency of a given antigen-specific T cell repertoire reproducibly generated by thymocyte selection is important as antigens with higher precursor frequencies can reach peak numbers more rapidly and provide better immune protection (27). Antigen-specific T cell repertoires with lower precursor frequencies will have a greater fold expansion, but will still not outnumber the higher precursor frequency repertoires (86). Based on this functional data, one could assume that the repertoires with larger precursor frequency have a higher affinity for pMHC as this would mean they have undergone less negative selection. Recent work supports this hypothesis as epitopes with lower precursor frequency demonstrate more similarity to mouse self-peptides as well as reduced pMHCII tetramer binding (27). Therefore, a balance exists between positive and negative selection and their preference to favor low- and high-affinity interactions between pMHC and TCR will ultimately determine the numbers and affinity of antigen-specific T cells.

All the advantages of TCRs with high affinity for pMHC would suggest they can easily outcompete the lower affinity T cells (5), but this does not seem to be occurring when the full affinity repertoire is analyzed (8, 9, 48). One caveat to many model systems is the focus on a single TCR and the use of different APLs to model the fate of polyclonal T cells in response to lower affinity antigens (7, 59, 87–89). In TCR-Tg mice, each T cell undergoes similar thymic selection mechanisms, and therefore possesses similar reactivity to antigen. Groups have shown different repertoires undergo different TCR selection and peripheral tolerance mechanisms, including negative selection, agonist selection, clonal diversion, and inhibitory molecule upregulation (65, 70, 74, 90, 91). For example, every OT-I TCR-Tg T cell will be positively and negatively selected by the same self-antigens, while in a polyclonal system, T cells with a range of affinities will recognize the OT-I cognate peptide (SIINFEKL) and will be positively and negatively selected by numerous different peptides. In the polyclonal setting, there will be a range of affinities for SIINFEKL and an accompanying range of tolerance mechanisms to control responses to this antigen. Therefore, a single TCR binding to different affinity pMHC complexes during selection is not the same as a polyclonal set of different TCRs binding to a set of pMHC. This distinction between effects at a clonal as opposed to a polyclonal level could alter functionality and impact interpretation on the role of lower affinity ligands during an immune response.

After primary antigen exposure and triggering of signaling cascades, division of CD4 and CD8 T cells will cause 100- to 1,000-fold expansion (61, 86, 92). Interestingly, low-affinity T cells are easily detectable throughout the response, signifying they are capable of expanding as well as high-affinity T cells (9, 48). Higher affinity CD8 TCR interactions cause asymmetric division that is associated with increased functionality of the proximal daughter cells (89). These CD8 T cells have similar initial rates of division, but eventually the highest affinity T cells maintain division while the lower affinity T cells begin to contract (7, 89). The contraction of lower affinity T cell is not due to increased death or lack of memory formation as a similar frequency of low-affinity pMHC primed CD8 T cells differentiates into memory T cells (88, 89). Along with differences in division rates, the migration kinetics of T cells are controlled by affinity, with the lower affinity APL stimulated T cells demonstrating increased numbers of TCR-Tg T cells in the blood at earlier time points (7). Studies in high- and low-affinity CD4 T cells demonstrates the time to the first division of high-affinity CD4 T cells is much faster than low-affinity cells, though after several divisions, the low-affinity cells reach the same absolute number as high-affinity T cells (67). Together, these data demonstrate low- and high-affinity T cells behave similarly during initial expansion, but most likely have roles in the immune response at distinct times and locations.

Evidence demonstrates T cell affinity controls effector and memory differentiation of antigen-specific populations. Using the OT-I CD8 T cell APL system, groups have demonstrated low-affinity priming generates a greater frequency of Eomes+ memory T cells (88). It was determined that TGF-βR expression, a negative regulator of T cells, is not downregulated in low-affinity T cell responses, creating a balance of the generation (IL-12R) and ablation (TGF-βR) of memory T cells (88). In CD4 T cells, TCR:pMHC affinity has been correlated with memory (6, 93, 94), T helper subset (T_H_1 vs. T_H_2), and T follicular helper (T_FH_) differentiation (92, 93, 95, 96) as well as prevention of exhaustion by chronic antigen exposure (97). Lower affinity TCR interactions have been shown to be biased to generate long-lived memory cells (6, 93, 94), while for T_H_1 vs. T_H_2 differentiation, greater strength of TCR stimulation increases the likelihood of T_H_1 differentiation (95, 96). For T_FH_ cells, increased and decreased TCR:pMHC affinity has been correlated with differentiation, thought to be due to TCR-dependent IL-2/IL-2R alterations (28, 92, 93). The finding that T_FH_ cells can differentiate from TCRs with low and high-affinity TCR:pMHC interactions is perplexing, but demonstrates that active mechanisms maintain the differentiation diversity of the low and high-affinity T cells. Nonetheless, comparing and contrasting the findings of CD4 and CD8 T cells demonstrates the complexity of each system, but also demonstrates unique roles and pathways for high- and low-affinity TCR:pMHC interactions to presumably maintain functional diversity.

The activation and regulation of metabolic pathways is essential for the initiation and maintenance of the immune response (98–101). In CD8 T cells, the TCR:pMHC interaction controls initial metabolic reprograming by upregulating IRF4 and Myc in a TCR:pMHC affinity-dependent manner (101). The transcription factors Myc and IRF4 coordinate the switch from fatty acid oxidation to aerobic glycolysis, which is essential for maintenance of the immune response (99, 101). Low-affinity TCR interactions led to less Myc and/or IRF4 expression, reducing the uptake of metabolic intermediates and changing the amount of T cell death during the response (99, 101). Therefore, TCR:pMHC affinity is necessary for instructing metabolic reprograming and a generating a greater functional response, but it is still unclear what function affinity-based metabolic reprograming plays for the differentiation and maintenance of low-affinity T cells.

## Maintenance of Affinity Diversity

The maintenance of clonotype diversity in the immune system is essential for the health of the organism (102). By maintaining clonotype diversity, TCR affinity diversity is also preserved, with a single epitope being recognized by multiple T cells to create a normally distributed TCR:pMHC affinity population. For each epitope, a range of T cell precursors exist, whose frequency is controlled by central tolerance mechanisms (27, 103). As the preimmune frequency for a single epitope approaches 0, the capacity of the repertoire to protect the host from this epitope diminishes (90, 104). This theoretical lower limit of T cells that can effectively protect the host is defined as a protecton and is dependent upon the size of the organism and the migration velocity of T cells (105). Larger organisms need a larger protecton because their bodies have a greater volume to patrol and protect against pathogen dissemination.

Along with the size of the organism, the protecton is dependent upon the amount of cross-reactivity between TCRs. To derive the level of cross-reactivity inherent to an individual’s TCRs, the entire number of antigen-specific T cells inclusive of lower affinity ones needs to be defined. Recent studies have identified T cells with cross-reactivity using multiple high-affinity-dependent methodologies, such as pMHC tetramers, but the rules regulating cross-reactivity are still being formulated (27, 104, 106, 107). Previous studies have suggested that the highest affinity T cells are the most cross-reactive as these cells have been shown to accept the most degeneracy in TCR:pMHC interaction and still function (108–110). However, single T cells can have both increased and decreased functional responses to peptides, meaning that just because a TCR binds to one pMHC with lower affinity, it cannot bind to another pMHC with higher affinity (106). Theoretically, a single TCR should possess a range of affinity for peptides presented by MHC, meaning that cross-reactivity is not unique to only high- or low-affinity TCRs. Groups have identified low-affinity T cells during the immune response, signifying these cells must be represented in the naïve mouse, yet these lower affinity T cells are currently not being included in the calculations (8, 9, 48). If inclusion of lower affinity T cells leads to a greater number of T cells in an antigen-specific repertoire, then the amount of cross-reactivity would correspondingly change (104). Therefore, protecton size and cross-reactivity calculations may be inaccurate due to the exclusion of these cells.

Low-affinity T cells are effective immune mediators and can have dominant roles in the immune response under specific conditions (62, 88, 111–113). When T cell clones are compared for their ability to cause autoimmunity, combat infection, or prevent tumors, low-affinity T cells are often comparable in accomplishing these tasks (22, 62, 97, 112, 113). For example, when a lower affinity CD8 T cell clone specific for an influenza antigen is transferred into a mouse expressing the antigen in a tumor, little immune response occurs (113). However, when this mouse is infected with influenza and/or given CD4 T cell help, the low-affinity T cells can respond with enhanced function (113). Along with influenza, groups have demonstrated adjuvants, such as CFA, MPL, and *Listeria monocytogenes*, can generate a larger population of low-affinity T cells (6, 112, 114, 115). Besides adjuvants, the form of antigen can control low-affinity T cell expansion as the use of protein antigen has been demonstrated to recruit more low-affinity T cells into the immune response (94). Why antigen and adjuvant influence the affinity diversity of the T cell response is still unclear, though these factors point to the type of antigen presenting cells (APCs) as a possible manipulator of low- and high-affinity T cell skewing. This suggests high- and low-affinity T cells may compete for TCR signaling, but mechanism, such as antigen processing and presentation, may maintain and influence the affinity diversity.

Alteration of APC by using different adjuvants or forms of antigen is one way to potentially alter T cell diversity, but can diversity of affinity be regulated in a T cell intrinsic fashion? As previously mentioned, the 2D affinity measurement by 2D-MP is a relative affinity, dependent unknown factors as well as the contact area restricting the receptor and ligand interactions. Our work reveals slightly different affinities (<10-fold) for thymocytes, peripheral naïve TCR-Tg T cells, and activated T cells demonstrating that the context of the membrane environments plays a role [(13) unpublished data]. Thymocytes, naïve, and activated T cells are different sizes, which may alter contact area between the T cell and RBC during the 2D-MP measurement. At the macroscopic level, the contact area difference between thymocytes and naïve T cells seems negligible, but in fact could result in differences as lymphocytes contain excess membranes, which is stored in ruffles and protrusions that could change during development and activation state (116). If there are differences in the ruffling of the membrane along with size differences, the membrane surface area in contact containing the TCR and pMHC could vary between different populations of cells. Of note, within a given population of cells, the surface area would be similar allowing for accurate affinity measures. These effects on membrane surface would also be predicted to influence lymphocyte function *in vivo*, which could be why 2D affinity so accurately predicts the level of functional response.

In addition, 2D affinities are dependent on the local membrane structure that is maintained by the actin cytoskeleton and controlled by the membrane lipid composition. During TCR activation, the actin cytoskeleton is remodeled (117). This cytoskeleton change could alter the 2D-MP affinity as the integrity of the membrane structure and orientation of the surface proteins will also fundamentally change. Inhibition of actin polymerization has been demonstrated to reduce 2D affinity as well as functional responses (12, 13). The actin inhibitors again demonstrate how 2D-affinity measurements accurately readout the functionality of the TCR interaction with pMHC (12, 15). A T cell could manipulate its 2D affinity through changes in the cytoskeletal support and protein attachment to actin. Alternative 2D affinity could be regulated to a degree by alterations in lipid content as CD4 T cell subsets contain differential organization of lipids (118, 119). Lipid composition has not been studied in relation to 2D affinity, but lipid order and organization has been demonstrated to be important in T cell functional responses, implicating the affinity may be different (118, 119).

Other surface proteins could influence the ability of the TCR to interact with pMHC and the 2D affinity. In the kinetic-segregation model of T cell activation, the size of the CD45 molecule regulates the interaction of TCR:pMHC, with its exclusion from the synapse a necessary step to initiate the T cell signaling cascade (51, 52). T cell expression of a smaller isoform of CD45 would reduce steric hindrance and could increase the TCR:pMHC affinity measured by 2D-MP. Therefore, when the 2D affinity is calculated in the context of the cellular membrane, multiple T cell intrinsic factors can tune its measured value. *In vivo*, this fine-tuning of 2D affinity could be envisioned as a mechanism allowing for small alterations in the likelihood of TCR engagement (affinity for pMHC) while maintaining the diversity of the immune response.

## Summary

Evidence demonstrates lower affinity T cells most likely have overlapping and distinct roles when compared to T cells with higher affinity interactions. Low-affinity T cells use much of the same signaling machinery for generating an immune response, but also must possess unique pathways or factors to sustain function and prevent excessive negative regulation during signaling. During differentiation, low-affinity T cells can again be found to have shared and unique roles when compared to higher affinity T cells. Low and high-affinity T cells must function together to efficiently generate a complete immune response and maintain the diversity of TCR affinity to efficiently protect the host.

## Conflict of Interest Statement

The authors declare that the research was conducted in the absence of any commercial or financial relationships that could be construed as a potential conflict of interest.
